# In Vitro Pharmacodynamics and Bactericidal Mechanism of Fungal Defensin-Derived Peptides NZX and P2 against *Streptococcus agalactiae*

**DOI:** 10.3390/microorganisms10050881

**Published:** 2022-04-22

**Authors:** Yankang Wu, Na Yang, Ruoyu Mao, Ya Hao, Da Teng, Jianhua Wang

**Affiliations:** 1 Gene Engineering Laboratory, Feed Research Institute, Chinese Academy of Agricultural Sciences, Beijing 100081, China; wykang123456@126.com (Y.W.); nana_891230@126.com (N.Y.); maoruoyu@caas.cn (R.M.); haoya@caas.cn (Y.H.); 2 Key Laboratory of Feed Biotechnology, Ministry of Agriculture and Rural Affairs, Beijing 100081, China

**Keywords:** *Streptococcus* *agalactiae*, antimicrobial peptides, NZX, P2, pharmacodynamics, bactericidal mechanism

## Abstract

(1) Background: Based on the hazard of *Streptococcus agalactiae* to human and animal health and the increasing drug resistance, it is urgent to develop new antimicrobial agents with high bactericidal activity and low drug resistance against *S. agalactiae*. This study aims to investigate in vitro pharmacodynamics and bactericidal mechanism of fungal defensin-derived peptides NZX and P2 against *S. agalactiae*. (2) Methods: Minimum inhibitory concentration (MIC) and mutant prevention concentration (MPC) were determined by broth dilution method and AGAR plate dilution method. Cell membrane integrity was determined by flow cytometer. Cell morphological changes were observed by scanning electron microscope (SEM) and transmission electron microscope (TEM). (3) Results: MIC values (NZX: 0.11 μM, P2: 0.91 μM) and MPC (NZX: 1.82 μM) showed their higher antibacterial activity and stronger inhibition ability of drug resistance mutation. The bactericidal mechanism was elucidated that P2 caused *S. agalactiae* ACCC 61733 cells to deform, bound to the cell wall, and perturbed cell membrane, resulting in K^+^ leakage, membrane hyperpolarization, ATP release, and reduced cell contents. Compared with P2, NZX focuses on the cell wall, and it bound to the cell wall causing cells boundary disappearance. (4) Conclusion: NZX and P2 are promising antimicrobial agents for streptococcicosis treatment.

## 1. Introduction

Fish and fisheries products are actually recognized as some of the healthiest foods on the planet. According to the statistics from The State of World Fisheries and Aquaculture 2020 of FAO (http://www.fao.org/3/ca9229en/ca9229en.pdf (accessed on 1 June 2020)), global aquaculture production increased to 82.1 million tons, and per capita, apparent consumption was 20.5 kg in 2018. However, with the increasing development of the scale and intensification of aquaculture, fish diseases caused by microbial pathogens more and more obviously appeared. *Streptococcus agalactiae* (*S. agalactiae*), as a zoonotic disease pathogen, can not only cause fish streptococcosis and dairy cow mastitis [1,2,3] but also colonizes the lower genital tract of 10–30% of women potentially causing sepsis and meningitis in their newborns [4,5], which can cause serious health hazards to animals and humans. Fish streptococcosis is a vital disease affecting many species of fish and is characterized by septicemia, meningoencephalitis, and exophthalmia [6]. *S. agalactiae* is the most prevalent pathogen among *Streptococcus* species and causes high morbidity and mortality, and its economic impact on the tilapia industry is greater than 1 billion USD, which has been a hindrance to tilapia farming worldwide [1,7,8]. Vaccination prevention and antibiotic treatment are primary measures for reducing tilapia damage caused by *S. agalactiae*. The function of vaccines can be exerted greatly by the precise diagnosis of epidemic strains and polyvalent vaccines because of the different serotypes and genetic profiles of *S. agalactiae* [6]. At present, the problem that antibiotic treatment has to face is that antimicrobial misuse to control bacterial infection and intensive culture of fishes has led to the emergency of multidrug-resistant isolates, as in the case of antibiotic treatment of tilapia infected with *S. agalactiae* [6,7,9]. Therefore, it is imperative to develop new antimicrobial agents with high bactericidal activity and low drug resistance against *S. agalactiae*.

Antimicrobial peptides (AMPs), as essential components in the innate immune system [10,11], have attracted considerable attention to be a new generation of antimicrobials to solve antibiotic resistance due to their broad antimicrobial spectrum, low toxicity, and low drug resistance [12,13]. The antimicrobial mechanism of AMPs is quite complex. Membrane permeabilization, as the primary mechanism of action, is widely accepted. Furthermore, AMPs interfere with intracellular target molecules by inhibiting the synthesis of critical proteins and the replication and transcription of DNA, and inhibit the synthesis of the cell wall by interfering with cell wall components or their precursors [14,15,16]. AMPs could cover the merits of antibiotics in disease treatment and vaccines in disease prevention, and avoid their shortcomings, such as high resistance and high variation in pathogens, and high residue in animals. AMPs, antibiotics, and vaccines could complement each other, and build an iron triangle of animal health care together to halt the spread of drug resistance [17,18]. Moreover, some literature reported that defensins, as a subcategory of AMPs, were involved in the treatment of infection-induced by *Streptococcus*, in which the C-terminal 15 amino acids of human beta-defensin-3 (HBD3-C15) inhibited the growth of the cariogenic pathogen *Streptococcus mutans* and its biofilm formation, and insect *Tribolium castaneum* defensin1 showed antibacterial activity in an in vitro blood-derived macrophages infection model of *Streptococcus pneumoniae* [19,20].

As the first fungal defensin, plectasin was isolated from the saprophytic ascomycete *Pseudoplectania nigrella*, which displays potent antibacterial activity against *Staphylococcus* and *Streptococcus* [21,22]. To further enhance the antibacterial activity of plectasin, the D9, M13, and K32 sites of plectasin were mutated, and the derivative peptide was named as NZX [23]. NZX showed superior antibacterial activity against clinical and multi-drug resistant strains of *Mycobacterium Tuberculosis* and *Staptococcus hyicus* in vitro and in vivo (mouse infection model) than rifampicin or ceftriaxone [23,24]. NZX also displayed intracellular bactericidal activity towards *M. tuberculosis* in primary macrophages and *S. hyicus* in Hacat cells [23,24]. Tenland et al. found that loading NZX into mesoporous silica particles increased mycobacterial killing in primary macrophages and preserved the activity to eliminate *M. tuberculosis* in a murine model [23].

Yang et al. used plectasin as the template and screened the homologous peptide P2 of plectasin in the fungal genome bank [25]. Compared with plectasin, P2 showed more than four-fold higher antibacterial activity towards *Staphylococcus aureus*, *Streptococcus pneumoniae*, and *Streptococcus suis* [25]. The mechanism of action of P2 against bacteria varies from cytoplasmic membrane permeabilization to interaction with intracellular genomic DNA; however, the degree of damage to different bacteria is different. P2 caused *Streptococcus dysgalactiae* cellular content leakage and cell shrinkage, and some *S. dysgalactiae* cells appeared to display a “ghosting” phenomenon, but *S. aureus* maintained cells integrity after treatment with P2 [25,26]. P2 also effectively inhibited early biofilm formation, eradicated mature biofilms, and even killed persisters of *S. dysgalactiae* and *S. aureus* [25,26].

NZX and P2 also have the characteristics of low hemolysis, no cytotoxicity, and high stability to pH and pepsin, which are beneficial to clinical application, particularly for oral or injection usages [24,25]. Additionally, the recombinant expression NZX and P2 in higher yield were successfully realized by the *Pichia pastoris* X-33 expression system, which will lay the foundation of their industrialized preparation and push them further toward clinical application [24,25].

Based on previous research on the highly expressing recombinant P2 and NZX and their antibacterial effects on multidrug resistance (MDR) *S. aureus* and *S. hyicus*, this study is trying to expand the research on the potential of P2 and NZX as antimicrobial agents to *S. agalactiae*. In this study, in vitro pharmacodynamics and bactericidal mechanism of NZX and P2 against *S. agalactiae* were elucidated for the first time.

## 2. Materials and Methods

### 2.1. Bacteria and Reagents

*S. agalactiae* ACCC 61733 (Ia, ST7) was obtained from the South China Sea Fisheries Research Institute of Chinese Academy of Fishery Sciences and stored at the Agricultural Culture Collection of China (ACCC) and was chosen as the target pathogen of the following study. *S. agalactiae* ATCC 13813 (II, ST310) was purchased from the American Type Culture Collection. *S. agalactiae* CAU-FRI 1 (Ia, ST103), CAU-FRI 2 (Ia, ST103), CAU-FRI 3 (Ia, ST103), and CAU-FRI 4 (Ia, ST103) isolated from bovine mastitis were obtained from the China Agricultural University. *S. agalactiae* PBSA0903 (Ia, ST7) isolated from tilapia was obtained from Hainan University. *Streptococcus* *dysgalactiae* CVCC 3938 was purchased from the China Veterinary Culture Collection Center (CVCC). NZX and P2 with purity over 90% were expressed by *P. pastoris* X-33 and purified based on our previous protocol [24,25]. All other chemical reagents used were of analytical grade.

### 2.2. Pharmacodynamics Evaluation of NZX and P2

#### 2.2.1. Minimal Inhibitory Concentration (MIC), Mutant Prevention Concentration (MPC), and Selection Index (SI) Determination

The MIC values of NZX and P2 were determined by the micro broth dilution method [27]. The test strains were diluted with tryptic soy broth medium (TSB) to 1 × 10^5^ CFU/mL after being cultured in TSB medium at 250 rpm and 37 °C until the mid-log phase. The cell suspensions (90 μL) and diluted test drug (10 μL) with the different final concentrations (0.0625–128 μg/mL) were added to a 96-well plate and incubated at 37 °C for 18–24 h. The MIC value is defined as the lowest concentration at which no bacteria grew. All tests were performed in triplicate.

The MPC of the test drug against *S. agalactiae* was measured by referring to the AGAR plate dilution method [28]. Based on the MICs of the test drug, the TSA plates with final concentrations ranging from 1× MIC to 128 μg/mL were prepared. *S. agalactiae* cells in mid-log phage were diluted to 3.0 × 10^10^ CFU/mL. A 100 μL of bacterial suspension was inoculated to each test drug plate and incubated at 37 °C. The MPC is defined as the concentration of sterile colony growth for 72 h. SI is the ratio of MPC to MIC [29].

#### 2.2.2. Time-Kill Curves Measurement

The time-killing kinetic curves determination was used to evaluate the bactericidal effect of NZX and P2 on *S. agalactiae* ACCC 61733 [30]. Briefly, the *S. agalactiae* cells were diluted to 1 × 10^5^ CFU/mL with TSB medium after being cultured in TSB medium at 37 °C until the mid-log phase and incubated with different concentrations (1×, 2×, or 4× MIC) of peptides at 250 rpm and 37 °C. A 100 μL of the sample was taken for colony counting on TSA plates at different time points (0–24 h). Florfenicol (Ff) and PBS were used as a positive control and negative control, respectively.

#### 2.2.3. Synergism and Post-Antibiotic Effect (PAE) of NZX and P2

The checkerboard method was applied to detect possible synergistic effects of peptides in combination with Ff [31]. The peptides and Ff were dispensed into 96-well cell culture plates at final concentrations ranging from 1/16 to 8× MIC in each well at 37 °C for 18 h. The MICs were measured according to the above MIC assay method. The combination effects were assessed by calculating the fractional inhibitory concentration index (FICI) of each combination. FICI = FIC of peptide + FIC of Ff, in which FIC of peptide = MIC of peptide in combination with Ff/MIC of peptide alone; FIC of Ff = MIC of Ff in combination with peptide/MIC of Ff alone. The interaction between peptides and Ff was evaluated according to the FICI: FICI ≥ 4 indicates antagonism, 1 < FICI ≤ 4 indicates indifference, 0.5 < FICI ≤ 1 indicates additivity, and FICI ≤ 0.5 indicates synergy.

The PAE of NZX and P2 against *S. agalactiae* ACCC 61733 was measured according to the previous method [32,33]. Briefly, after being treated with 1×, 2×, and 4× MIC NZX or P2, *S. agalactiae* cells (1 × 10^8^ CFU/mL) were diluted 1000 times with TSB medium and incubated at 37 °C and 250 rpm. At the end of each exposure time point, 100 μL of the sample was taken for colony counting on TSA plates. Ff and PBS were used as a positive control and negative control, respectively. The PAE was calculated using the following formula: PAE (h) = T − C, T: the time required for the number of colonies in the test culture to increase by 10 times above the number of colonies at 0 h, C: corresponding time for the untreated group.

### 2.3. Effect of Peptides on the Cell Membrane

#### 2.3.1. FACS Analysis of Cell Membrane Integrity

A membrane permeability experiment was used to study the effects of NZX and P2 on the integrity of *S. agalactiae* ACCC 61733 cell membrane. *S. agalactiae* ACCC 61733 cells in mid-log phage (1 × 10^8^ CFU/mL) were incubated with NZX or P2 at a final concentration of 4× MIC at 37 °C for 30 min and 120 min, respectively. After treatment with peptides, the bacteria were washed twice with PBS and stained with propidium iodide (PI). Finally, a flow cytometer (FACS Calibur, BD, USA) was applied to determine the fluorescence [34].

#### 2.3.2. K^+^ Leakage

*S. agalactiae* ACCC 61733 cells in mid-log phage (1 × 10^5^ CFU/mL) were exposed to the test drug at a final concentration of 5× MIC and incubated at 37 °C. A 200 µL bacterial solution was taken at different time points and centrifuged. A 100 µL of supernatant was taken and diluted 1000 times with sterile ultrapure water. Finally, the concentration of K^+^ was detected by atomic absorption spectrometer (ContrAA 700 Analytik Jena, Jena, Germany) [16]. Triton X-100 and ultra-pure water were used as positive control and blank control, respectively.

#### 2.3.3. ATP Release Reaction

The ATP release was determined using the BacTiter-GloTM Microbial Cell Viability Assay kit (Promega). *S. agalactiae* ACCC 61733 cells in mid-log phage (1 × 10^5^ CFU/mL) were treated with different concentration test drugs ranging from 1 to 32× MIC at 37 °C for 1 h. The BacTiter-Glo™ Reagent was added, and the luminescence was recorded with the help of a multifunctional microplate reader (Infinite M1000, Tecan, Switzerland). The test was conducted in triplicate. As a positive control, 2% Triton X-100 was used. The fold reduction of ATP was calculated using the following formula: Fold reduction of ATP = 1 − [(L_treat_ − L_media_)/(L_control_ − L_media_], in which, L_treat_ is the luminescence of cells treated with test drug, L_media_ is the luminescence of TSB without cells, and L_control_ is the luminescence of untreated cells [35].

#### 2.3.4. Membrane Potential

The 3,3-dipropylthiadicarbocyanine iodide (DiSC_3_(5)) assay was used to investigate the effects of NZX and P2 on the membrane potential of *S. agalactiae* ACCC 61733 cells [35]. *S. agalactiae* ACCC 61733 cells in mid-log phage (1 × 10^8^ CFU/mL) were mixed with DiSC_3_(5) at a final concentration of 0.5 mM for 30 min. Different final concentration peptides (0.5, 1, 2, 4, and 8× MIC) were added, and the fluorescence intensity was determined with excitation wavelength (620 nm) and emission wavelength (670 nm) using a multifunctional microplate reader (Infinite M200, TECAN (Shanghai) Trading Co., Ltd., Shanghai, China). Two percent Triton X-100 and PBS were used as positive and blank control, respectively. The experiment was performed in triplicate.

### 2.4. Effect of Peptides on Cell Wall

#### 2.4.1. Scanning Electron Microscopy (SEM) Observations

SEM was applied to more intuitively observe the effects of NZX or P2 on *S. agalactiae* cell surface morphology. After being treated with 4× MIC NZX or P2 for 2 h, *S. agalactiae* ACCC 61733 cells were fixed at 4 °C overnight in 2.5% glutaraldehyde, dehydrated with gradient ethanol series (50–70–85–95% × 2–100% × 2, 15 min/time), and dried with CO_2_. The samples were sputtered using platinum and observed using an SEM (QUANTA200, FEI, Philips, The Netherlands) [25].

#### 2.4.2. Transmission Electron Microscopy (TEM) Observations

*S. agalactiae* ACCC 61733 cells were treated with 4× MIC NZX or P2 for 2 h and fixed at 4 °C overnight in 2.5% glutaraldehyde. After postfixed in 1% osmium for 1 h and dehydrated with gradient ethanol series (50–70–85–95–100% × 3, 15 min/time), *S. agalactiae* cells were embedded in epoxy resin, sliced, and stained with 1% uranium acetate. TEM (JEM1400, JEDL, Tokyo, Japan) was applied to observe the *S. agalactiae* cell morphology and intracellular changes [25].

#### 2.4.3. Super-Resolution Microscopy (SRM) Observations

The localization of NZX and P2 in *S. agalactiae* ACCC 61733 cells was observed using SRM [36]. After being incubated with 4× MIC FITC-NZX or FITC-P2 at 37 °C for 30 min, *S. agalactiae* cells were stained with DAPI (10 μg/mL) and PI at 4 °C for 15 min and washed with PBS. The sample was mixed with an anti-fluorescence quenching agent and then was added to polyTM microscope slides, sealed with cover glass, and observed using SRM (N-SIMS, Nikon, Japan). The *S. agalactiae* cells untreated with FITC-NZX or P2 were used as the blank control.

#### 2.4.4. Bactericidal Effect of NZX and P2 on Quiescence/Division Period Bacteria

*S. agalactiae* ACCC 61733 cells in mid-log phage were diluted to (1 × 10^8^ CFU/mL) with PBS and TSB broth, respectively, and treated with NZX, P2, Ff, penicillin, gentamicin, vancomycin, streptomycin, nisin and bacitracin at final concentrations of 2× and 4× MIC at 37 °C for 2 h, respectively. The sample (100 μL) was taken for colony counting on TSA plates.

#### 2.4.5. Interaction with Lipid II

NZX and P2 were exposed to 5 mM pyrophosphate, sodium pyrophosphate, Nα, Nε-Diacetyl-Lys-D-Ala-D-Ala (dKAA), and D-glutamate at 37 °C for 1 h, respectively [16,37]. The MICs were measured as the above MIC assay method.

## 3. Results

### 3.1. Pharmacodynamics Evaluation

#### 3.1.1. MIC, MPC, and SI of NZX and P2 against *S. agalactiae* ACCC 61733

As shown in Table 1 and Table 2, NZX and P2 exhibited more potent antibacterial activity towards *Streptococcicosis* (including *S. agalactiae* and *S. dysgalactiae*) with MIC values of 0.06–0.22 μM and 0.46–0.91 μM, respectively, than antibiotic Ff with MIC values of 2.80–5.59 μM. The MPC of NZX, P2, and Ff against *S. agalactiae* ACCC 61733 was 1.82 μM > 29.2 μM, and >357.5 μM, respectively. Therefore, NZX had a small range of mutant selection window (MSW) with 0.11–1.82 μM and Ff had a wide range of MSW with 5.59 -> 357.5 μM. The SI of NZX was 16, which was lower than those of P2 (>32) and Ff (>64). It suggested that NZX has a lower probability to lead the enrichment of drug-resistant mutant subspecies than P2 and Ff.

#### 3.1.2. Time-Killing Curves of NZX and P2

**Figure 1:**
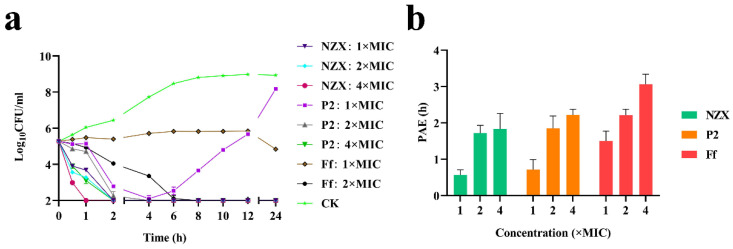
Time-killing curves and PAE. (**a**) Time-killing curves of NZX and P2 against *S. agalactiae* ACCC 61733. The *S. agalactiae* cells were treated with NZX and P2 (1×, 2×, or 4× MIC) at 250 rpm and 37 °C for 2 h, respectively. A 100 μL of the sample was taken for colony counting on TSA plates at different time points of 0–24 h. (**b**) PAEs of NZX and P2 to *S. agalactiae* ACCC 61733. Results are given as the mean ± SEM (n = 3). CK was the untreated group.

The time-killing curves showed (Figure 1a) that *S. agalactiae* ACCC 61733 was significantly decreased after treatment with NZX and P2 for both time- and concentration-dependence. *S. agalactiae* cells were killed completely within 2 h at 1–4× MIC NZX and 4× MIC P2, and after they were treated with 1× and 2× MIC P2, the bacteria were killed completely within 4 h, but bacteria regrowth occurred after 4 h at 1× MIC P2. Additionally, 1× MIC Ff could only inhibit *S. agalactiae* growth, and the *S. agalactiae* cells were killed completely within 6 h at 2× MIC Ff.

#### 3.1.3. Synergism and PAE of NZX and P2

The combination of NZX and P2 with Ff showed a FICI value was 1.0625 (Table 3). According to the synergy index, it determined that NZX and P2 had indifference effects with Ff. Under the concentration of 1×, 2× and 4× MIC, the PAE values of NZX against *S. agalactiae* ACCC 61733 were 0.57, 1.72, and 1.83 h, and those of P2 were 0.71, 1.85, and 2.22 h. The PAE values of Ff were 1.5, 2.21, and 3.06 h, respectively (Figure 1b). The results indicated that the PAE values of NZX, P2, and Ff were concentration-dependent.

### 3.2. Effect of Peptides on the Cell Membrane

#### 3.2.1. Integrity of Bacterial Membrane

**Figure 2:**
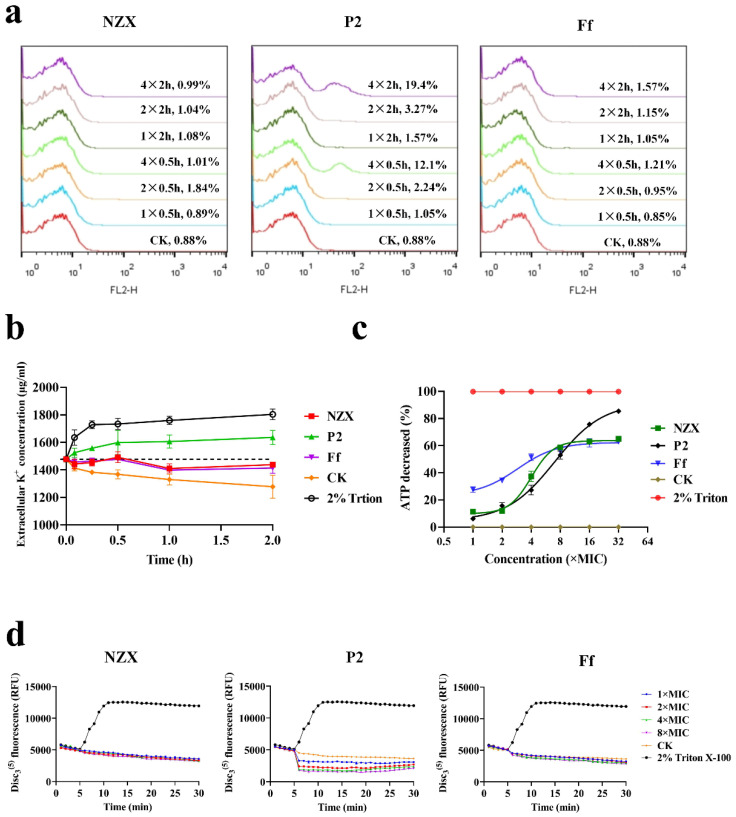
Effects of NZX and P2 on the cell membrane: (**a**) Effects of NZX and P2 on integrity of *S. agalactiae* ACCC 61733 cells membrane. The membrane penetration rates of NZX and P2 to *S. agalactiae* cells were detected by flow cytometer under the concentration of 1×, 2×, and 4× MIC for 30 min and 2 h, respectively. (**b**) Changes in the concentration of extracellular K^+^ of *S. agalactiae* ACCC 61733 cells caused by NZX and P2. The concentration of extracellular K^+^ of *S. agalactiae* was detected by atomic absorption spectrometer under treatment with 5× MIC NZX and P2 for 0–2 h, respectively. (**c**) ATP release of *S. agalactiae* ACCC 61733 cells caused by NZX and P2. ATP content of *S. agalactiae* cells was measured using a multifunctional microplate reader after treatment with 1–16× MIC NZX and P2 for 1 h, respectively. (**d**) Changes of membrane potential of *S. agalactiae* ACCC 61733 cells caused by NZX and P2. Membrane potential of *S. agalactiae* ACCC 61733 cells was determined by multifunctional microplate reader under the concentration of 0.5–8× MIC NZX and P2 for 30 min. CK was the untreated group.

The effect of NZX and P2 on the cell membrane integrity of *S. agalactiae* ACCC 61733 was determined by a flow cytometer. The percentage of PI-stained *S. agalactiae* cells was only 0.88% in the untreated group (Figure 2a). After treatment with different concentrations of NZX and Ff for 0.5 and 2 h, the percentages of PI-stained cells were 0.89–1.84% (NZX) and 0.85–1.57% (Ff), which showed no difference compared with the untreated group. The percentages of PI-stained cells treated with 1×, 2×, and 4× MIC P2 for 0.5 h were 1.05%, 2.24%, and 12.1%, respectively. Comparably, after treatment with the same concentration of P2 for 2 h, percentages of PI-stained cells were 1.57%, 3.27%, and 19.4%, respectively. The results indicated that NZX did not destroy the membrane of *S. agalactiae*, but P2 increased the bacteria membrane permeability in a concentration-dependent manner.

#### 3.2.2. K^+^ Leakage

Cell membrane integrity can be further identified by measuring the K^+^ leakage of cells, which own to the imbalance of K^+^ inside and outside the cell once the cell membrane is destroyed [38]. In the untreated group, the extracellular K^+^ concentration gradually decreased to maintain bacteria life activities with the continuous reproduction of the cells (Figure 2b). The bacterial cultures exhibited significant K^+^ leakage after being exposed to P2 within 5 min compared with the untreated group, which displayed a similar trend to the positive control (Triton X-100). However, after treatment with NZX and Ff, the extracellular K^+^ concentration did not change significantly, indicating that the bacterial growth had been inhibited, but the bacterial membrane remained intact. This result was consistent with the integrity of the bacterial membrane.

#### 3.2.3. ATP Release Reaction

ATP content is closely related to the transmembrane proton dynamic potential energy. Luminescent microbial cell viability assays determined the effects of NZX and P2 on ATP efflux of *S. agalactiae* ACCC 61733 cells, as shown in Figure 2c, when *S. agalactiae* cells were exposed to 16× and 32× MIC P2, the percentages of ATP release were 75.7% and 85.3%, which were higher than those of NZX (63% and 65.2%) and Ff (62% and 62.9%). The result suggested that P2 significantly affected the transmembrane proton dynamic potential energy of *S. agalactiae*.

#### 3.2.4. Membrane Potential

The ability of NZX and P2 to interfere with the electrochemical potential of the cytoplasmic membrane of *S. agalactiae* ACCC 61733 was measured by the voltage-sensitive dye DiSC_3_(5). The Triton X-100 changed the potential difference between the inner and outer membrane immediately with a significant increase in fluorescence intensity, resulting in depolarization. After treatment with P2, the fluorescence decreased in a concentration-dependent manner (Figure 2d). The result showed that P2 could perturb the cellular membrane, causing dissipation of membrane potential, and induce hyperpolarization of the membrane. However, the fluorescence intensity was consistent with that of the control group after *S. agalactiae* cells were exposed to NZX and Ff, which indicated that the membrane potential remained constant. The results further proved that P2 and NZX had different bactericidal mechanisms.

### 3.3. Effect of Peptides on Cell Wall

#### 3.3.1. SEM Observations

**Figure 3:**
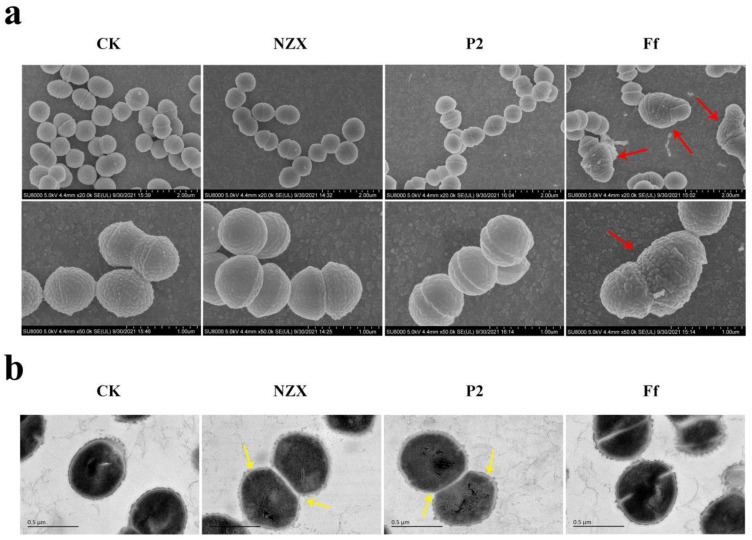
Effects of NZX and P2 on the cell morphology and ultrastructure: (**a**) SEM observations of *S. agalactiae* ACCC 61733 cells treated with NZX and P2. The *S. agalactiae* cells were exposed to 4× MIC NZX and P2 for 2 h, respectively; The red arrows in the Ff treatment group indicate that the cell surface was swollen and deformed, and normal division could not be completed. (**b**) TEM observations of *S. agalactiae* ACCC 61733 cells treated with NZX and P2. The *S. agalactiae* cells were exposed to 4× MIC NZX and P2 for 2 h, respectively; The yellow arrows in NZX and P2 treatment groups indicate that the cell wall became thin, blurred and even the cell’s boundary disappeared. CK was the untreated group.

The microscopic morphology changes of *S. agalactiae* ACCC 61733 were observed using SEM. The untreated *S. agalactiae* cells had intact morphology and a smooth surface (Figure 3a). When *S. agalactiae* cells were exposed to 4× MIC Ff, the cell surface was swollen and deformed, and some filamentous adhesions were observed on the surface of cells, and cells could not divide normally. After treatment with NZX and P2, there were no significant morphological changes in the cells, except the decreased number of bacteria.

#### 3.3.2. TEM Observations

The effects of peptides on the ultrastructure of *S. agalactiae* ACCC 61733 were observed using TEM. The untreated *S. agalactiae* had normal cell morphology and an intact cell wall, and the electrical density of bacteria was evenly distributed (Figure 3b). After exposure to NZX, the cell wall of *S. agalactiae* became incomplete and blurred, and the cell’s boundary completely disappeared near the membrane of cells that were about to divide. After treatment with P2, the same changes happened to the *S. agalactiae* cell wall, but cell contents were reduced, and the cytoplasmic electron density became uneven. However, Ff caused abnormal cell division, indicating different action modes among peptides NZX, P2, and Ff.

#### 3.3.3. SRM Observations

**Figure 4:**
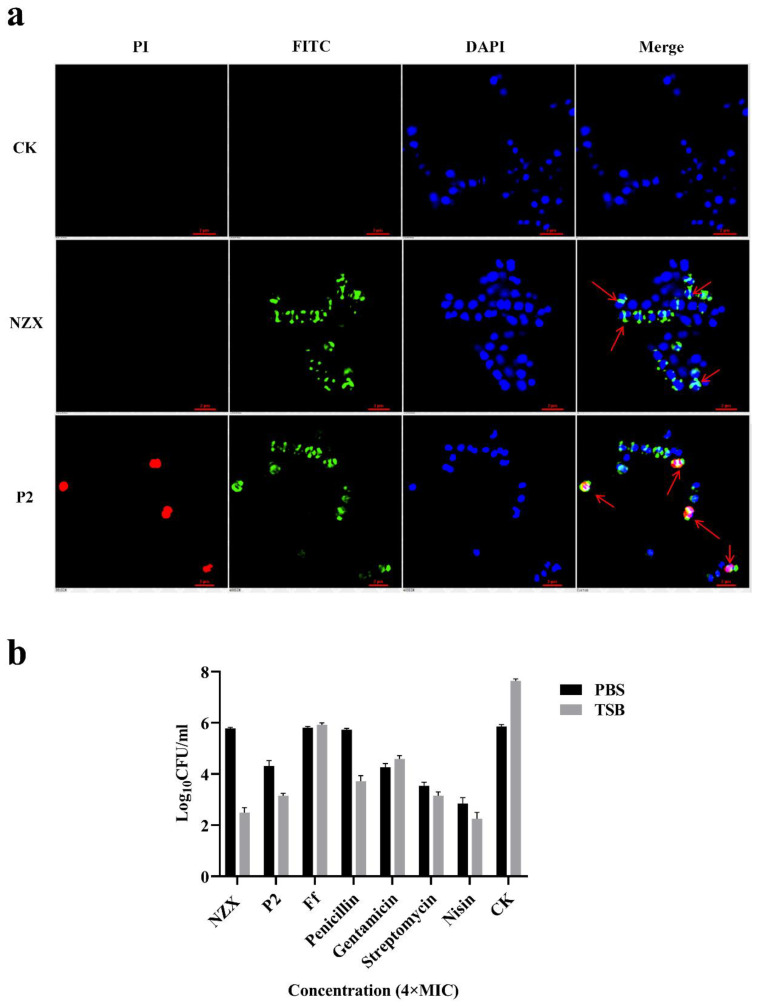
Localization of FITC-labeled peptides on cells and bactericidal ability of peptides on quiescence and division period bacteria: (**a**) SRM observations of localization of FITC-labeled NZX and P2 on *S. agalactiae* ACCC 61733 cells. The *S. agalactiae* cells were treated with 4× MIC FITC-labeled NZX and P2 for 30 min, respectively. (**b**) The bactericidal ability of NZX and P2 to *S. agalactiae* cells on quiescence and division period (suspended in PBS and TSB broth). The *S. agalactiae* cells were treated with NZX and P2 (2× and 4× MIC) for 2 h, respectively. CK was the untreated group.

To explore the action targets of NZX and P2 on *S. agalactiae* ACCC 61733, the positions of FITC-labeled NZX and P2 on *S. agalactiae* cells were observed by SRM. As shown in Figure 4a, only blue fluorescence (DAPI) derived from the nucleus was detected in the control group. After treatment with FITC-NZX and FITC-P2, green fluorescence was located on the cell wall of the dividing daughter cells. This indicated that NZX and P2 were bound to the cell wall of the dividing cells. In addition, red fluorescence (PI) was detected in the FITC-P2 group but not observed in the FITC-NZX group, which showed that P2 increased the permeability of the cell membrane, resulting in PI penetrating the plasma membrane and binding with the nucleic acid. However, NZX displayed a different action mechanism with P2, and it did not disrupt the cell membrane.

#### 3.3.4. Bactericidal Effect of NZX and P2 on Quiescence/Division Period Bacteria

Antimicrobial drugs that have bactericidal effects on breeding bacteria usually target the cell wall of bacteria during the breeding period, blocking the synthesis of peptidoglycan [16,37,39]. To further prove that the targets of NZX and P2 are related to the cell wall or not, the bacteria were retained at different growth stages. In the PBS buffer, the bacteria have no nutrient intake and cannot complete division, while in the nutrient medium (TSB), the bacteria metabolize and proliferate normally. Based on this principle, the bactericidal ability of these two peptides was tested in TSB and PBS. As shown in Figure 4b, when *S. agalactiae* ACCC 61733 cells were suspended in PBS, the bacteria number remained constant after exposure to NZX. However, when NZX and *S. agalactiae* cells were incubated in TSB, the bactericidal ability was significantly improved, and the number of viable colonies was significantly decreased by 5.6 log_10_ compared with the control check (CK) group. The result was similar to the penicillin-treated group, which targeted cell walls (3.93 log_10_ reductions in TSB). After treatment with P2, the number of viable colonies was 1.56 log_10_ reductions in PBS and 4.5 log_10_ reductions in TSB. While under the same initial colony number, the difference in bactericidal ability between the two environments is only 1.15 log_10_. It determined that the cell wall was one of the targets of P2, but not the only one. Gentamicin, streptomycin, and nisin perform the bactericidal activity with a non-cell wall-targeting mechanism, and they displayed similar bactericidal ability in PBS and TSB. This further illustrated the different bactericidal mechanisms between NZX and P2.

#### 3.3.5. Interaction with Lipid II

Lipid II is the site of action of many drugs targeting the cell wall [40]. In order to determine whether lipid II plays a role in the cell wall-related mechanism of action of NZX and P2, MIC assay was administrated in the presence of dKAA (mimic the D-alanyl-D-alanine, part of the pentapeptide in lipid II), pyrophosphate, and D-glutamate to observe the competitive inhibition of peptides activity. The mechanism of plectasin is to bind to the pyrophosphate and D-glutamate on lipid II to interfere with the normal synthesis of the cell wall, and vancomycin binds to D-alanyl-D-alanine [16,37]. As shown in Table 4, the MIC value of positive control Van increased 32 times in the presence of dKAA, while the antibacterial activity of NZX and P2 did not change in the presence of dKAA, pyrophosphate, and D-glutamate, respectively, which preliminarily indicated that the target of NZX and P2 was not on these three parts of lipid II.

## 4. Discussion

*S. agalactiae* is a zoonotic pathogen that can cause infection in pregnant women, neonatal bacteremia and meningitis, cow mastitis, and tilapia streptococcus disease [41]. Antibiotics were used as traditional therapeutic drugs, but resistance was developed due to long-term or improper use, especially for β-lactam antibiotics [42], which significantly restricts the solution of public health and animal husbandry problems caused by *S. agalactiae*. Therefore, the development of novel potential antibacterial drugs is urgently needed. Plectasin had a specific bactericidal effect on Gram-positive bacteria [21,43] and low immunogenicity, which opened the door for the application of fungal defensins. In this study, plectasin-derived peptide NZX and homologous peptide P2 showed superior antibacterial activity towards *S. agalactiae* than antibiotic Ff. MSW and SI are pharmacodynamic indexes for evaluating the ability of antibiotics to induce bacterial resistance [44]. NZX has a lower MPC value (1.82 μM), SI value (16), and a smaller range of MSW (0.11–1.82 μM) than those of Ff (MPC value > 357.5 μM, SI value > 64, MSW: 5.59 -> 357.5 μM) (Table 2), which could effectively prevent the selection and enrichment of drug-resistant mutants. Time-killing curves showed that the killing rates of NZX and P2 against *S. agalactiae* ACCC 61733 were higher than that of Ff (Figure 1a). However, the PAE of NZX and P2 was lower than that of Ff under the same MIC (Figure 1b), NZX and P2 could take advantage of rapid sterilization in a short time. The structural parameters of AMPs are crucial to the activity. Studies have shown that introducing a positive charge into antimicrobial peptides will increase antibacterial activity, while introducing a negative charge will decrease antibacterial activity [45]. The Lys residue at position 32 was replaced by Arg residue with higher charge density in NZX [46]. Therefore, the high antibacterial activity of NZX may be related to the characteristic that the isoelectric point and charge number of NZX are higher than P2.

Plectasin and its derivative NZ2114 are non-lytic membrane AMP, which could bind to lipid II to block bacterial cell wall biosynthesis [16]. Although NZX and P2 belong to the fungal defensins family and are highly homologous with plectasin, they display different antibacterial mechanisms. According to the result of membrane integrity (Figure 2a), the PI-stained percentage of *S. agalactiae* ACCC 61733 cells were 12.1% and 19.3% after being treated with P2 at 2× MIC and 4× MIC for 2 h, which indicated that P2 caused the permeability of the bacterial membrane in a concentration-dependent manner, the result was consistent with the previous study [25]; however, the bacteria maintained membrane integrity with a few PI-stained after treatment with NZX. Moreover, K^+^ leakage and membrane potential disorder assays further proved that P2 perturbed the membrane and caused the K^+^ leakage and potential imbalance (Figure 2b,d). The cell membrane potential increases the negative value of the inner membrane, at this time, K^+^ keeps flowing out of the cell, resulting in decreased fluorescence intensity and the cell membrane hyperpolarization [47,48]. The dissipation of membrane potential and proton gradient was accompanied by ATP leakage [49]. ATP is a donor to maintain normal cell energy metabolism and exchange, which reflects the number of surviving cells [50]. When the drug depletes the membrane potential and proton gradient, ATP synthesis is blocked in the absence of proton motive potential energy driving, which can explain the reason that P2 displayed a higher inhibition rate on ATP (32× MIC, 85.3%) than that of NZX (32× MIC, 65.2%) at the same MIC in this study (Figure 4a). Therefore, the membrane permeabilization and depolarization were related to the mode of action of P2. However, the NZX treatment group did not cause the change in membrane permeability and K^+^ leakage and maintained the membrane potential balance, which demonstrated that the mechanism of NZX did not target the cell membrane.

In addition, the morphology and ultrastructure of *S. agalactiae* and fluorescence localization of peptides determined that the target location of NZX and P2 (green fluorescence) was at the cell wall during bacterial division and caused thinning of the cell walls and a blurred boundary, and the red fluorescence (PI-stained) detected in the cell proved that P2 caused the change of membrane permeability of *S. agalactiae* cells, which was different with NZX, the result was consistent with the FACS results. It is preliminarily speculated that the cell wall may be one of the targets of NZX and P2. In the previous studies, the antibacterial activity of telavancin decreased in a nutrient-poor environment (PBS) due to the bacteria stopping dividing and drugs losing the target on lipid II [37,39]. In this study, NZX and P2 lost or decreased bactericidal ability in PBS, while bactericidal ability was significantly improved in TSB, which was similar to penicillin and telavancin. The bacteria survive in isotonic PBS, but they lack the nutrients required for cell wall synthesis and growth. NZX and P2 lost the specific cellular target to bind to the cell wall without the new cell wall synthesis. Plectasin had been proven to exert an antibacterial function by binding Lipid II. The amino acid residues of positions 2, 3, 4, and 37 at the N-terminal of plectasin formed hydrogen bonds with the pyrophosphate moiety of lipid II, His18, and D-γ-glutamate of lipid II formed a salt bridge, which ultimately blocks cell wall synthesis [16]. The plectasin-derived peptide NZX and homologous peptide P2 retain the amino acid sites on binding Lipid II (Table 5). Vancomycin, another glycopeptide drug that acts on cell walls, blocks cell wall synthesis by binding D-Ala-D-Ala on Lipid II. However, the effect of parts or analogue of lipid II (dKAA, pyrophosphoric acid, and D-glutamate) on the antibacterial activities of the NZX and P2 did not display competitive inhibition. Therefore, it was speculated that the target of NZX is located on the cell wall, P2 interacted with the bacterial cell wall, and perturbed permeability and potential of the cell membrane. The different modes of action of NZX and P2 may be due to the degree of α-helix, although NZX and P2 have the typical CSαβ conformation, including an α-helix (NZX: residues 12–20 and P2: residues 13–21), an antiparallel β-sheet, and three disulfide bonds, P2 showed a greater degree of α-helix (36.4%) than that of NZX (18.3%) in anionic SDS solutions of the membranes-mimetic environment [24,25], the greater α-helical structures are more easily permeabilized the bacterial membranes [51]; therefore, this may explain the reason that P2 displayed the permeability on the cell membrane, NZX had no effect on the cell membranes. However, it needs further studies to reveal the detailed targets of NZX and P2 on *S. agalactiae*. These results suggest that NZX and P2 have the potential to treat *S. agalactiae*, which effectively reduces the loss of *S. agalactiae* infection to the tilapia industry.

## 5. Conclusions

**Figure 5:**
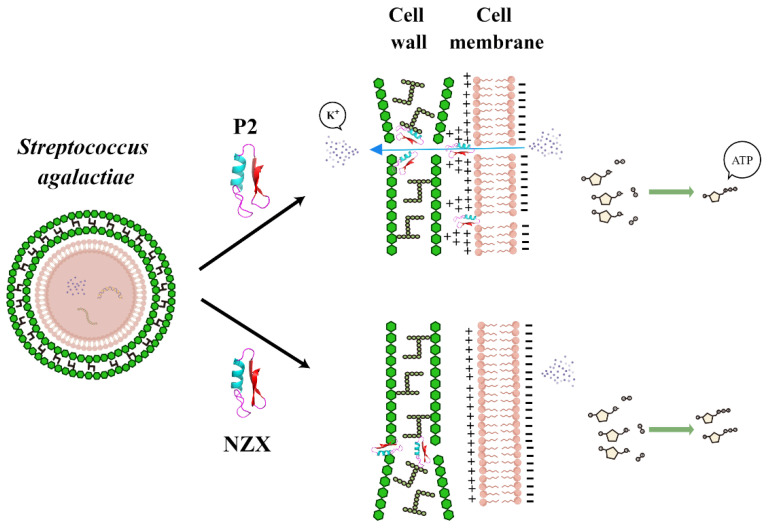
Mode of actions of NZX and P2 against *S. agalactiae* ACCC 61733.

In this study, in vitro pharmacodynamics and bactericidal mechanisms of fungal defensin-derived peptides, NZX and P2 against *S. agalactiae* ACCC 61733 were systemically studied for the first time. Compared with Ff, NZX and P2 showed a more efficient and faster antibacterial activity towards *S. agalactiae*, and NZX also exhibited a lower probability to lead the enrichment of drug-resistant mutant subspecies to *S. agalactiae* ACCC 61733 than P2 and Ff. Amino acid changes among parent peptide plectasin and its derived peptides lead to their changes in α-helix, charge number, and so on, and then they display different mechanisms of action toward *S. agalactiae* cells. P2 had a dual mechanism of action of interfering with the cell wall and disrupting the cell membrane. P2 could cause *S. agalactiae* cells to deform, bind to the cell wall, and destroy the cell membrane, resulting in K^+^ leakage, membrane hyperpolarization, ATP release, and reduction of cell contents (Figure 5). Compared with P2, the mechanism of action of NZX focused on the cell wall, NZX bound to the cell wall causing cells boundary disappearance, and its effect on the cell membrane is only shown in transmembrane proton dynamic potential energy. These results indicate that NZX and P2 are promising antimicrobial agents to treat *S. agalactiae* infection, especially for fish streptococcicosis treatment.

## Figures and Tables

**Table 1:** The MIC of peptides and Ff.

Strain	MIC					
	NZX		P2		Ff	
	μg/mL	μM	μg/mL	μM	μg/mL	μM
*S. agalactiae* ATCC13813	0.50	0.11	4	0.91	1	2.80
*S. agalactiae* CAU-FRI 1	0.25	0.06	2	0.46	1	2.80
*S. agalactiae* CAU-FRI 2	0.50	0.11	2	0.46	2	5.59
*S. agalactiae* CAU-FRI 3	0.50	0.11	4	0.91	2	5.59
*S. agalactiae* CAU-FRI 4	0.50	0.11	4	0.91	2	5.59
*S. agalactiae* PBSA0903	0.50	0.11	4	0.91	1	2.80
*S. dysgalactiae* CVCC 3938	1	0.22	2	0.46	1	2.80

**Table 2:** The MIC, MPC and SI of NZX and P2 against *S. agalactiae* ACCC 61733.

Variety	MIC		MPC		SI
	μg/mL	μM	μg/mL	μM	
NZX	0.5	0.11	8	1.82	16
P2	4	0.91	>128	>29.2	>32
Ff	2	5.59	>128	>357.5	>64

**Table 3:** Synergism of peptides with Ff.

Combination	Variety	MICa (μg/mL)	MICc (μg/mL)	FIC	FICI
NZX-Ff	NZX	0.5	0.5	1	1.0625
	Ff	2	0.125	0.0625	
P2-Ff	P2	4	0.5	1	1.0625
	Ff	2	0.125	0.0625	

Note: MICa, the MIC of variety drug alone; MICc, the MIC of the most effective combination.

**Table 4:** The effect of lipid II analogues on the antibacterial activity of peptides.

Variety	None	dKAA(D)	Sodium Pyrophosphate (S)	D-Glutamate (D-G)
	MIC_A_ (μg/mL)	MIC_A+D_ (μg/mL)	MIC_A+S_ (μg/mL)	MIC_A+D-G_ (μg/mL)
NZ2114	0.5	0.5	0.5	0.5
NZX	0.5	0.5	0.5	0.5
P2	4	4	4	4
VAN	2	64	2	2

**Table 5:** Amino acid sequences and physicochemical properties of peptides [12,24,25].

Peptides	Sequences	Similarity (%)	Length (Amino Acid)	MW (Da)	pI	Charge (+)	GRAVY	Hydrophobicity
Plectasin	GFGCNGPWDEDDMQCHNHCKSIKGYKGGYCAKGGFVCKCY	100.0	40	4407.9	7.77	1	−0.695	0.353
P2	GFGCNGPWDEDDM**K**CHNHCKSIKGYKGGYCA**SA**GFVCKCY	92	40	4380.9	7.77	1	−0.573	0.366
NZX	GFGCNGPW**S**EDD**I**QCHNHCKSIKGYKGGYCA**R**GGFVCKCY	92	40	4390.0	8.3	2	−0.578	0.385

## Data Availability

Data is contained within the article.
